# Supplementary material for: Metabolic Syndrome in Obese Children—Clinical Prevalence and Risk Factors
Source: Int J Environ Res Public Health. 2021 Jan 25;18(3):1060. doi: 10.3390/ijerph18031060 (PMC7908375; doi:10.3390/ijerph18031060)
Supplement: Supplementary file 1 [file ijerph-18-01060-s001.pdf]

Supplement

Table 1. Mean glucose, triglycerides (TG) and high density lipids (HDL) concentration in study population

	Girls n=275		Boys n=316		
Concentration	Mean	SD	mean	SD	p
Glucose [mg/dl]	91,07	8,51	93,22	9,58	0,003
TG [mg/dl]	92,96	48,52	82,9	44,64	0,001
HDL[mg/dl]	45,62	9,21	47,4	10,31	0,031

$p<0.05$ Chi square test analysis.

Table 2. Comparison of glycemia, TG and HDL abnormalities between boys and girls.

	All the children		Girls		Boys		
	n	%	n	%	n	%	P
glycemia<100 mg/dl	521	88,2	250	90,9	271	85,8	0,053
glycemia>=100mg/dl	70	11,8	25	9,1	45	14,2	
TG <150 mg/dl	538	91	249	90,5	289	91,5	0,699
TG >=150mg/dl	53	9	26		27	8,5	
HDL>40mg/dl	431	72,9	197	71,6	234	74,1	0,510
HDL<40mg/dl	160	27,1	78	28,4	82	25,9	

$p<0.05$ Kruskal-Wallis test analysis

Table3. Dinner consumption in the study population.

Dinner consumption min 2 hr before sleeping	MetS+		MetS–		MetS+/–		MetS+ vs MetS – vs MetS +/-	MetS+&MetS +/- vs MetS –	MetS+ vs MetS– &MetS +/-
							(p)		
All the children	n	%	n	%	n	%			
No	27	35.5	63	25.9	98	40.3	0.033	0.009	0.418
Yes	49	64.5	180	74.1	174	59.7			
Girls	n	%	n	%	n	%			

No	11	36.7	38	30.1	37	31.1	0.763	1.000	0.516
Yes	19	63.3	88	69.8	82	68.9			
Boys	n	%	n	%	n	%	0.001	0.0001	0..611
No	16	34.7	25	20.2	61	41.7			
Yes	30	65.3	99	79.8	85	58.3			

p<0.05 Kruskal-Wallis test analysis

. Table 4. Physical performance analysis.

Physical performance	MetS+		MetS–		MetS+/–		MetS+ vs MetS – vs MetS +/-	MetS+& MetS +/- vs MetS –	MetS+ vs MetS– &MetS +/-
							(p)		
All the children	n	%	n	%	n	%	0.043	0.088	0.044
Poor	24	31.5	32	13.1	50	18.3			
Normal	52	68.5	211	86.9	222	81.7			
Girls	n	%	n	%	n	%	0.103	0.154	0.601
Poor	5	16.7	6	5.1	18	14.3			
Normal	25	83.3	113	94.9	108	85.7			
Boys	n	%	n	%	n	%	0.034	0.403	0.015
Poor	21	45.6	25	20.2	31	21.2			
Normal	25	54.4	99	79.8	115	78.8			

p<0.05 Kruskal-Wallis test analysis

.
